# Eye-Tracking-Based Digital Therapeutic for ADHD in Children: Development and Preliminary Evaluation of an Adaptive Learning Guide

**DOI:** 10.3390/healthcare14111486

**Published:** 2026-05-27

**Authors:** Seon-Chil Kim, Sun-Young Lee, Sang-Woo Lee

**Affiliations:** 1 Department of Biomedical Engineering, School of Medicine, Keimyung University, 1095 Dalgubeol-daero, Daegu 42601, Republic of Korea; 2 Biomedical Engineering Laboratory, Department of Biomedical Engineering, School of Medicine, Keimyung University, 1095 Dalgubeol-daero, Daegu 42601, Republic of Korea; syl982008@gmail.com; 3 Department of Rehabilitation Science, College of Rehabilitation Sciences, Daegu University, 201 Daegudae-ro, Gyeongsan-si 38453, Republic of Korea; lsw3545@inthetech.co.kr

**Keywords:** ADHD, digital therapeutics (DTx), eye tracking, adaptive intervention, selective attention

## Abstract

**Highlights:**

**What are the main findings?**

- Eye-tracking-based adaptive digital therapeutic content significantly improved attentional performance (Attention Composite) over 4 weeks.
- Selective improvements were observed in cognitive domains related to response inhibition and visual search (Stroop Color–Word, Interference, and Symbol Search), with limited effects on basic processing speed measures.

**What are the implications of the main findings?**

- Real-time gaze-based feedback may enhance the effectiveness of digital therapeutics by detecting and correcting attentional disengagement during task performance.
- Eye-tracking-based metrics may serve as meaningful process-based indicators that complement standardized neuropsychological assessments in ADHD interventions.

**Abstract:**

Background: This study evaluated the preliminary clinical effects of an eye-tracking-based adaptive learning guide in a self-administered digital therapeutic for children with ADHD. Methods: In this prospective, randomized, parallel-group pilot trial, 40 children with ADHD (aged 6 to under 12 years) were allocated to an experimental group receiving eye-tracking-based adaptive digital therapeutic content or a control group receiving conventional digital therapeutic content, both combined with ongoing pharmacological treatment, for four weeks. Results: The Attention Composite significantly increased from 47.63 at Week 1 to 52.14 at Week 4, with attention-sensitive tasks showing steeper improvement trajectories than standard tasks. Changes in the Attention Composite were positively correlated with CAT score changes (r = 0.519, *p* = 0.0047). Significant between-group differences were observed in the Stroop Color–Word, Stroop Interference, and K-WISC-V Symbol Search measures, but not in the Word, Color, or Coding measures. Conclusions: These findings suggest that eye-tracking-based adaptive guidance may selectively improve attentional performance and cognitive control in children with ADHD, though larger-scale confirmatory studies are warranted.

## 1. Introduction

Attention-deficit/hyperactivity disorder (ADHD) is one of the most common neurodevelopmental disorders in childhood and adolescence and is characterized by persistent inattention, hyperactivity, and impulsivity, leading to difficulties in academic performance and daily functioning [1]. Children with ADHD often demonstrate deficits in selective and sustained attention, response inhibition, and self-regulation, which disrupt learning processes [2,3]. They are also easily distracted by external stimuli during learning tasks [4]. Consequently, ADHD may result in significant learning difficulties during critical developmental periods [5].

ADHD symptoms extend beyond a short attention span and are closely associated with executive functions that support stable attention to target stimuli and goal-directed behavior during task performance [6]. Therefore, interventions should address not only outcomes but also how attention is maintained, diverted, and restored during task performance. In this context, non-pharmacological approaches using digital technologies have gained increasing attention [7]. Computer-based cognitive training programs and digital therapeutics (DTx) offer advantages such as standardized repetitive training, improved accessibility, adaptive difficulty, enhanced engagement, and automatic performance tracking. Some studies have reported improvements in attention and working memory [8,9].

Pharmacological treatment effectively reduces the core symptoms of ADHD and is known to alleviate symptoms primarily by modulating the dopaminergic and noradrenergic systems in the brain [10]. However, although medication may be effective for the short-term reduction in inattentive and impulsive symptoms, limitations may remain in achieving long-term improvements in functional domains such as sustained attentional engagement, behavioral self-regulation, and social adaptation in everyday settings [11]. Therefore, digital interventions may serve as adjunctive approaches that complement pharmacological treatment by providing repeated cognitive training, real-time feedback, and enhanced treatment engagement.

Despite these promising features, evidence for the effectiveness of digital interventions remains inconsistent. The extent to which performance gains translate into improvements in clinical symptoms, real-world learning behaviors, and everyday functioning requires careful interpretation [12,13]. These findings suggest that effectiveness depends not only on content quality but also on sustained user engagement during task performance, highlighting the importance of embedded feedback mechanisms.

Although several digital therapeutic and cognitive training studies have reported improvements in attentional performance and working memory in children with ADHD, the reported effects have been variable across studies, with some studies demonstrating only modest effect sizes or limited transfer to real-world functioning [14,15]. In addition, inconsistent adherence and limited monitoring of attentional engagement during self-administered use have been identified as important limitations affecting intervention effectiveness [16,17].

Self-administered digital interventions for children with ADHD have strong practical value, as they can be used repeatedly in home or everyday settings without direct supervision from clinicians, teachers, or therapists [18]. However, this autonomy may also reduce learning effectiveness, as children with ADHD are prone to attentional disengagement. Without immediate redirection from caregivers, children may appear to engage with content while attending to peripheral stimuli, or they may respond mechanically while their gaze is disengaged from the screen. Under such circumstances, session completion may not reflect meaningful learning. Nevertheless, many programs emphasize outcome-based metrics, such as accuracy, reaction time, persistence, and completion rates, whereas few systems detect attentional disengagement during task performance and provide real-time intervention [19,20]. Eye-tracking offers a promising solution by providing an objective, continuous indicator of attentional focus [21] and enabling precise measurement of learning processes and attentional allocation [22].

Children with ADHD may exhibit atypical visual fixation, oculomotor inhibition, and visual control toward target stimuli, suggesting that gaze data sensitively reflect attentional characteristics [23]. Therefore, eye-tracking can function not only as a post hoc assessment tool but also as a real-time process-based indicator of attentional state. When integrated with response rules within digital content, gaze data may enable a closed-loop system that redirects attention when it deviates from task-relevant areas. In educational technology and multimedia learning, visual signaling and attention-guidance strategies that direct gaze toward relevant information have been widely demonstrated as effective [24].

Previous studies have shown that modifying gaze allocation can improve comprehension and memory. However, this work has primarily focused on educational content for typically developing learners [25]. Consequently, research on the application, implementation, and empirical validation of such approaches in self-administered digital content for children with ADHD remains limited. Existing evidence highlights that the effectiveness of digital interventions depends on sustained engagement during task performance [26,27,28] and that eye-tracking can serve as an objective indicator of real-time attentional state in children with ADHD [29,30]. Embedding an eye-tracking-based adaptive learning guide in self-administered DTx and systematically evaluating its effectiveness may support the development of more effective interventions for this population.

This study developed digital content that detects attentional disengagement in real time during self-administered task performance in children with ADHD and delivers visual and auditory stimuli as adaptive interventions. When gaze deviates from the area of interest for a predefined duration or repetitive exploration of task-irrelevant regions is detected, the system automatically provides attentional prompts or pauses the task to redirect attention. This approach differs from previous studies by utilizing eye-tracking not merely as a measurement tool but as an adaptive intervention mechanism through real-time feedback. Furthermore, the study goes beyond comparisons of accuracy or completion rates by analyzing process-based performance indicators such as on-task gaze ratio, fixation duration within the area of interest, attention re-engagement time, omission errors, and reaction time variability. Through this approach, the study seeks to elucidate the mechanisms underlying learning effects during digital therapeutic interventions.

This study pursued three objectives. First, it aimed to demonstrate that decreased concentration and attentional disengagement during self-administered use represent critical practical challenges that may limit the effectiveness of DTx for ADHD. Second, it sought to extend the application of eye-tracking technology from a diagnostic or observational tool to an interventional mechanism providing immediate learning support. Third, it aimed to integrate clinical intervention research with established multimedia learning theories, to refine design principles for digital content for children with ADHD and evaluate their effectiveness. Additionally, the study proposes that self-administered DTx for children with ADHD may evolve beyond simple repetitive training tools into adaptive systems that respond to moment-to-moment attentional states and provide automated learning support.

Based on these considerations, we hypothesized that the eye-tracking-based adaptive learning guide would improve attentional performance and reduce attentional disengagement during self-administered digital therapeutic use in children with ADHD. We further expected that attention-sensitive tasks would demonstrate greater improvement trajectories than standard tasks and that gaze-based performance metrics would be associated with standardized neuropsychological measures of attention.

## 2. Materials and Methods

### 2.1. Study Design and Participants

**Figure 1:**
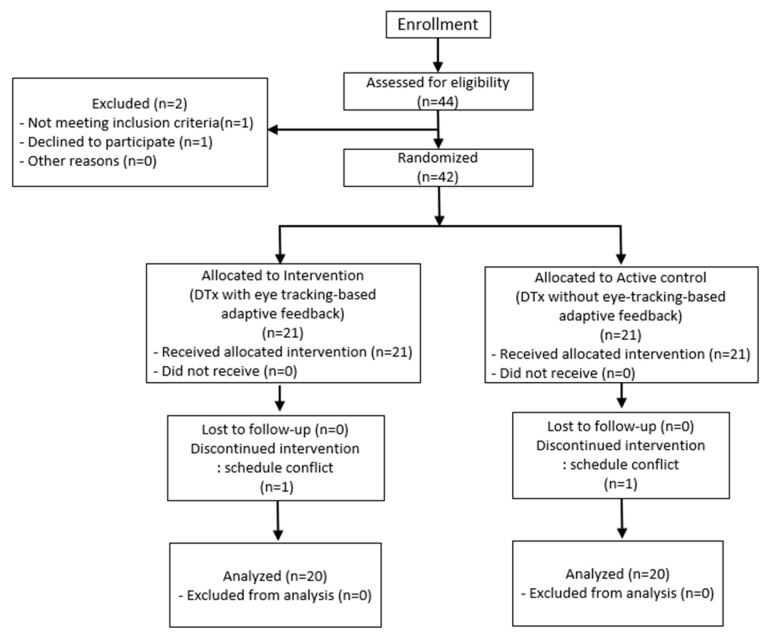
Flow diagram of study participants.

This study was a prospective, randomized, parallel-group pilot clinical trial to evaluate the effectiveness of eye-tracking-based interventional digital therapeutic content for children with ADHD [31]. The study was conducted over approximately four months, from 26 May 2025, to 30 September 2025, at the Department of Psychiatry of K University Hospital. The 4-week intervention period was selected based on precedents established in comparable ADHD digital therapeutic trials [9,32], the feasibility constraints of conducting a pilot study in young children, and the study’s primary focus on detecting preliminary changes in process-based attentional metrics rather than long-term clinical outcomes. Figure 1 illustrates the overall study flow, including participant enrollment, randomization, allocation to intervention and active control groups, follow-up, and final analysis.

Participants were children aged 6 to under 12 years diagnosed with ADHD by a board-certified psychiatrist according to the Diagnostic and Statistical Manual of Mental Disorders, Fifth Edition (DSM-5) [33]. The inclusion criteria were as follows. First, written informed consent was obtained from legal guardians and children provided assent. Second, participants were able to understand the study procedures and perform the required tasks according to the investigator’s instructions. Third, participants had intellectual functioning within the normal range based on screening assessments and were capable of performing the digital content tasks. Eligibility was determined through comprehensive evaluation, including clinical interviews conducted by a psychiatrist, medical history review, and psychological assessment results.

We excluded children with neurodevelopmental, psychiatric, or neurological conditions other than ADHD that could have significantly affected study outcomes, those with physical limitations that could have interfered with the operation of digital devices, and children with significant motor impairments, including seizure disorders. Color vision deficiencies that could have affected performance on color-based tasks and having family members from the same household already enrolled in or currently participating in the study were also exclusion criteria. Additionally, children deemed unsuitable for study participation at the investigator’s discretion were excluded.

Considering the pilot nature of the study and recruitment feasibility, the target sample size was set at 40 participants. An a priori sensitivity analysis was conducted using G*Power version 3.1.2 [34]. With a two-tailed significance level of α = 0.05 and statistical power (1 − β) of 0.80, the minimum detectable effect size for independent group comparisons was approximately d = 0.96. In an analysis of covariance (ANCOVA) model assuming a correlation of r = 0.60 between baseline and post-intervention scores, an effect size of approximately d = 0.77 was detectable. Accordingly, the study was interpreted as exploratory, aiming to identify effect direction, feasibility, and preliminary effect size estimates rather than confirm efficacy.

A total of 44 children were screened for eligibility. One participant who did not meet the inclusion criteria and one who declined participation were excluded. The remaining 42 participants were randomly assigned to the experimental or control group in a 1:1 ratio. Randomization was performed using a computer-generated randomization sequence by an independent researcher who was not involved in outcome assessment or intervention delivery. Allocation concealment was maintained using sequentially numbered sealed envelopes until participant enrollment was completed. Due to the nature of the intervention, participant blinding was not feasible. The experimental group received eye-tracking-based adaptive digital therapeutic content (EYAS-Focus DTx, Indertech Co., Ltd., Daegu, Republic of Korea, 2025) combined with standard pharmacological treatment, whereas the control group received conventional digital therapeutic content (NeuroWorld DTx, Woorisoft Co., Ltd., Daegu, Republic of Korea, 2025) with standard pharmacological treatment. During follow-up, one participant from each group withdrew because of scheduling conflicts. A total of 40 participants (20 per group) were included in the final analysis.

Pharmacological treatment was permitted only if maintained prior to study participation. Initiation of new ADHD medications during the study period was not allowed, and the permitted medications were limited to methylphenidate and atomoxetine formulations. All medications remained within the prescribed range established before study enrollment [35]. All participants were maintained on stable pharmacological treatment throughout the study period, and medication regimens were managed consistently across both groups.

This study was conducted in accordance with ethical guidelines approved by the Institutional Review Board. To minimize potential adverse effects associated with digital device use, such as headache, fatigue, dizziness, or frustration, session duration was limited to less than 30 min, and adverse events were continuously monitored.

### 2.2. Assessment Tools and Procedures

The assessments consisted of baseline evaluations conducted before the intervention and post-intervention evaluations performed after the 4-week intervention period. Baseline measures included demographic information, clinical characteristics, ADHD symptom severity, and neuropsychological assessments, including the Stroop Color and Word Test and the Korean Wechsler Intelligence Scale for Children—Fifth Edition (K-WISC-V). During the intervention, session-level performance data generated by the digital therapeutic device were also collected [36,37]. Post-intervention assessments repeated the same neuropsychological tests to evaluate intervention effects. Assessments were administered in a fixed order. At baseline, the CGI-S was administered first, followed by the Stroop Color and Word Test, CAT (Computerized Attention Test) and the K-WISC-V processing speed subtests. Post-intervention assessments were conducted in the same order, excluding the CGI-S.

In the present study, outcome-based measures referred to final performance outcomes such as accuracy, response time, and standardized neuropsychological assessment results, whereas process-based measures referred to real-time gaze- and performance-related indicators generated during task execution.

#### 2.2.1. Clinical Severity Assessment

Baseline clinical severity was evaluated using the Clinical Global Impression–Severity (CGI-S) scale. The CGI-S provides a global rating of current symptom severity and has been extensively used in psychiatric research, including ADHD studies [38]. In this study, it was used to confirm baseline clinical comparability between the groups and to describe overall symptom severity.

#### 2.2.2. Stroop Color and Word Test

Selective attention and response inhibition were assessed using the Stroop Color and Word Test. This well-established test measures the ability to inhibit automatic responses and allocate attention to task-relevant stimulus attributes [39,40]. It includes three conditions: word reading (Word), color naming (Color), and color–word interference (Color–Word). In the Word condition, participants read color–words as quickly as possible. In the Color condition, they named the ink color of the stimuli. In the Color–Word condition, incongruent stimuli required participants to inhibit the automatic reading response and instead respond to the actual ink color.

The following indices were derived: word, color, color–word, and interference scores. The color–word and interference scores were primary indicators of response inhibition and cognitive control. Raw scores were converted to T-scores (mean = 50, standard deviation = 10) based on normative data for statistical analysis [41].

#### 2.2.3. Korean Wechsler Intelligence Scale for Children—Fifth Edition

Processing speed-related subtests from the K-WISC-V were administered to assess attention, visual search, and processing speed. The Coding and Symbol Search subtests, which constitute the Processing Speed Index (PSI), were included in the analysis [42]. The Coding subtest required rapid and accurate transcription of symbols within a limited time, reflecting visual search ability, visuomotor coordination, attention, and processing speed. The Symbol Search subtest required rapid identification of target symbols within a set, thereby assessing visual discrimination, visual search, and processing speed [43]. Standardized scores from both subtests were used to compare pre- and post-intervention changes.

#### 2.2.4. Eye-Tracking-Based Performance Measures

Eye-tracking-based metrics generated during device use were collected to evaluate changes in attentional regulation. For each session, two primary indicators—hit efficiency and screen exit ratio—were calculated. Hit efficiency is a metric defined in this study to reflect the accuracy and efficiency of responses to target stimuli during task performance, whereas the screen exit ratio represents the proportion of time during which gaze or attention deviates from the task screen within a session. These metrics were derived from established concepts in eye-tracking research, including response efficiency and off-task gaze behavior [44]. The screen exit ratio was reverse-coded so that higher values indicated better performance.

To minimize the effects of scale differences and outliers between the two indicators, robust Z-score standardization based on the median and interquartile range was applied. Subsequently, the session-level Attention Composite raw score was calculated as the mean of the two standardized indicators and transformed to a 0–100 scale to facilitate interpretation. Equal weighting was applied to prevent disproportionate influence of either indicator. Hit efficiency and screen exit ratio were conceptualized as reflecting two conceptually distinct and complementary dimensions of attentional regulation—task response efficiency and attentional disengagement, respectively. Equal weighting was applied to prevent either dimension from disproportionately influencing the composite. However, the optimal weighting scheme has not been empirically validated, and data-driven approaches should be explored in future studies with larger samples.

As the Attention Composite was developed as a continuous process-based performance indicator for this study, no predefined threshold or cutoff values were established. The score is intended to reflect relative within-individual change in attentional engagement over the intervention period rather than to serve as a diagnostic classification criterion.

#### 2.2.5. Eye-Tracking Acquisition, Calibration, Preprocessing, and System Reliability

The eye-tracking system used in this study was designed to monitor attentional engagement and disengagement during self-administered digital therapeutic task performance rather than to perform ophthalmologic-grade fixation analysis. Gaze estimation was implemented using a front-facing RGB camera integrated into the tablet device. Facial and iris landmarks were extracted using a Mediapipe-based face mesh model consisting of 478 landmarks, including iris center points and eye contour landmarks. Relative gaze coordinates were estimated by calculating the positional relationship between iris center points and predefined eye-boundary landmarks.

Before each intervention session, a calibration procedure was conducted using predefined on-screen reference targets positioned at the center and directional screen locations. Participants were instructed to visually fixate on each calibration target to generate individualized gaze-coordinate mapping parameters. Recalibration was additionally performed when substantial gaze drift, unstable landmark detection, or repeated tracking instability was observed during task execution. To improve calibration reliability, intervention sessions were performed using a seated posture at a relatively consistent viewing distance with the tablet device positioned in landscape orientation.

All sessions were conducted under stable indoor lighting conditions. Excessive backlighting, strong screen reflections, and abrupt environmental illumination changes were minimized to improve facial landmark detection stability and reduce gaze-estimation noise associated with RGB camera-based tracking.

To reduce noise associated with excessive head movement, blinking, temporary eye closure, and transient facial tracking instability commonly observed in children with ADHD, several preprocessing procedures were applied before gaze-based metric extraction. Frames with unstable facial landmark detection, implausible gaze-coordinate fluctuations, temporary eye closure, or transient facial tracking loss were automatically excluded from valid gaze analysis. Gaze coordinates detected outside the predefined screen boundary were classified as off-screen events rather than valid gaze engagement signals.

The present study focused on detecting sustained attentional engagement within predefined task-relevant areas of interest (AOIs) rather than performing classical fixation-event analysis. Therefore, valid gaze engagement was operationally defined as continuous detectable gaze presence within the AOI across consecutive valid frames corresponding to a minimum temporal duration of approximately 200 ms. Brief transient gaze fluctuations below this threshold were not considered stable attentional engagement. Adaptive guidance was triggered when gaze deviation from the predefined AOI persisted beyond the predefined temporal threshold or when repetitive task-irrelevant scanning patterns were consecutively detected.

Internal technical validation was conducted under controlled viewing conditions using predefined on-screen target coordinates. Spatial estimation error was calculated based on the Euclidean distance between predefined target locations and estimated gaze coordinates. Under stable head-position conditions, the system demonstrated an approximate screen-level spatial error of approximately 20 mm, which was considered sufficient for AOI-level attentional engagement monitoring in the present study. Task-relevant AOIs were intentionally designed to occupy sufficiently large display regions to improve robustness against gaze localization variability. Based on the implemented digital therapeutic task design, the smallest AOIs ranged from approximately 55–60 mm in width (approximately 22–25% of screen width), which exceeded the estimated spatial gaze error (~20 mm) by approximately 2.7–3-fold in linear dimensions. Because attentional engagement was determined using sustained gaze presence across consecutive valid frames (~200 ms threshold) rather than single-frame gaze localization, transient spatial localization variability was expected to have limited influence on attentional-state detection and AOI classification. In children with ADHD, who tend to exhibit more frequent head movements and attentional shifts than typically developing peers, gaze estimation noise may be elevated, which should be considered a limitation when interpreting the reliability of attentional detection in this population [23,29]. Across all recorded intervention sessions, approximately 14.7% of gaze frames were excluded because of unstable landmark detection, blinking, temporary signal loss, or excessive motion-related artifacts. The remaining valid gaze samples were included in the session-level Attention Composite analysis. The system was designed for robust AOI-level attentional engagement monitoring in ecologically realistic self-administered DTx environments rather than high-resolution ophthalmologic eye-movement quantification.

### 2.3. Digital Content Types

The digital content consisted of a tablet-based therapeutic program designed to train children with ADHD in selective attention, sustained attention, visual search, and response inhibition [32,45]. Both groups had identical therapeutic targets, task structures, difficulty levels, and usage time limits. The primary difference was the provision of real-time learning guidance based on eye-tracking.

**Figure 2:**
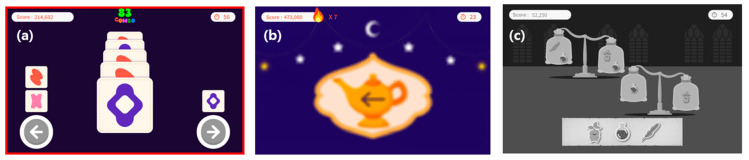
Examples of eye-tracking-based adaptive learning guidance provided when gaze deviation was detected. (**a**) Visual border highlighting to redirect attention to the task area. (**b**) Background blurring to enhance focus on task-relevant stimuli. (**c**) Temporary grayscale screen effect to facilitate attentional reorientation.

The experimental group received eye-tracking-based adaptive digital therapeutic content. A front-facing camera-based module estimated gaze position and analyzed fixation within the area of interest (AOI) in real time. Each session began with a calibration procedure to improve gaze detection accuracy. During task performance, when gaze deviated from the primary AOI for a predefined duration or repeated exploration of task-irrelevant areas was detected, the system delivered stepwise learning guidance according to predefined rules. This guidance consisted of visual outline highlighting, temporary blurring, and temporary grayscale modulation to redirect attention to task-relevant stimuli (Figure 2).

Among the ten digital therapeutic tasks, three were designed as attention-sensitive tasks responsive to changes in attentional state. These tasks required target detection, distractor inhibition, and sustained visual search, enabling subtle attentional changes to be reflected in behavioral performance metrics. Accordingly, gaze-based indicators (hit efficiency and screen exit ratio) were expected to show greater changes in these tasks than in others.

The attention-sensitive content in the experimental group was designed to evaluate the effectiveness of eye-tracking-based adaptive learning guidance. These tasks imposed high demands on target detection and created conditions in which attentional disengagement could occur readily. Tasks requiring rapid target identification were designed to produce larger changes in hit efficiency, whereas environments with competing distractors were expected to reflect changes in the screen exit ratio. In addition, tasks requiring responses within limited time windows were included to capture changes in attention control under time pressure.

**Figure 3:**
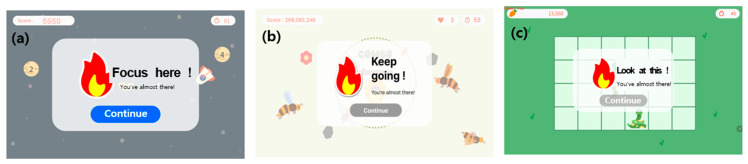
Examples of message-based attentional guidance provided when gaze deviation was detected during digital therapeutic tasks. (**a**) Focus reminder message. (**b**) Motivational prompt to encourage task persistence. (**c**) Task reorientation message.

In these attention-sensitive tasks, when gaze deviation from the task-relevant area was detected, adaptive learning guidance, such as task-relevant stimulus messages and temporary task pauses, was provided to redirect attention to the task (Figure 3). In the experimental condition, real-time gaze monitoring was additionally used to detect attentional disengagement, and gaze-based adaptive feedback was provided to facilitate task re-engagement during gameplay.

**Figure 4:**
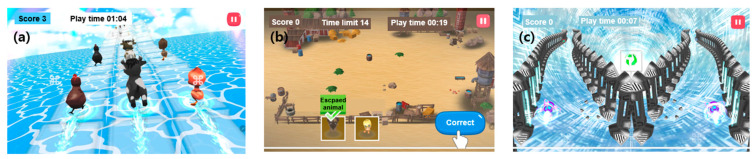
Types of control digital therapeutic tasks used in the study. (**a**) Selective attention training task. (**b**) Visual search training task. (**c**) Response inhibition training task.

The control group received conventional digital therapeutic content. Although the control content maintained the same task types and difficulty structure as the experimental condition, it did not include real-time adaptive feedback or gaze-based attentional cueing. Task difficulty was adjusted according to predefined performance criteria, including response accuracy and task completion performance. When participants demonstrated sustained successful performance, task difficulty gradually increased by modifying stimulus complexity, distractor density, or response speed requirements. Conversely, repeated attentional disengagement or poor performance triggered maintenance or reduction in difficulty levels. This design enabled evaluation of the additional effects of eye-tracking-based learning guidance rather than task structure alone (Figure 4).

All participants used the digital therapeutic content for four weeks. To minimize fatigue and frustration associated with digital device use, each session lasted less than 30 min. Session-level performance logs and eye-tracking-based metrics generated during the intervention were automatically recorded and analyzed after study completion.

### 2.4. Analysis Methods

Statistical analyses were performed using SPSS Statistics version 23.0 (IBM Corp., Armonk, NY, USA) and Python 3.10 (Python Software Foundation, Wilmington, DE, USA). All tests were two-tailed, and the significance level was set at *p* < 0.05. Continuous variables were reported as mean ± standard deviation or median with interquartile range, and categorical variables as frequencies and percentages. Normality was assessed using the Shapiro–Wilk test and Q–Q plots [46].

Baseline group comparability was assessed using Fisher’s exact test for categorical variables and independent *t*-tests for continuous variables when normality assumptions were met; otherwise, nonparametric tests were used [47]. Primary analyses compared intervention effects between the groups. The primary outcome was the Attention Composite score at the end of the 4-week intervention. Secondary outcomes included Stroop indices (color–word and interference scores) and K-WISC-V processing speed subtests (Coding and Symbol Search). Between-group differences in post-intervention scores were evaluated using analysis of covariance (ANCOVA) with the baseline values as covariates to adjust for minor baseline differences and improve statistical efficiency relative to change-score comparisons [48].

Temporal changes in session-level data were analyzed using linear mixed-effects models (LMMs) [49]. The dependent variable was the session-level Attention Composite score. Fixed effects included time (week, 1–4), group (experimental vs. control), and their interaction (week × group). To account for individual differences in baseline performance levels, a random intercept for each participant was included, and random slopes were added as needed based on model fit and convergence. Models were estimated using restricted maximum likelihood (REML). LMMs account for correlations in repeated measures while allowing the use of all available data despite some missing values [50].

Missing data handling differed by analysis set. The primary efficacy analysis used a full analysis set including participants who completed at least one intervention session and one post-intervention assessment. A per-protocol sensitivity analysis, including participants who completed the intervention as planned, was also conducted. Repeated session-level data were analyzed using LMMs, and missing data at single time points were handled after examining missingness patterns and applying appropriate methods.

Effect sizes were reported according to the statistical method. Partial eta squared (η^2^) was reported for ANCOVA, standardized effect sizes for between-group mean differences, and estimated regression coefficients with 95% confidence intervals for LMM. Effect sizes were interpreted as small, medium, or large based on established guidelines [51]. In addition, to evaluate the convergent validity of eye-tracking-based metrics, change scores (Δ values) from Week 1 to Week 4 were calculated, and Pearson correlation analyses were conducted with Stroop and processing speed measures. The 95% confidence intervals (CIs) for the correlation coefficients were estimated using 5000 bootstrap resamples [52].

## 3. Results

### 3.1. Participants Characteristics

Participant characteristics are presented in Table 1. The experimental group included 17 boys and 3 girls, and the control group consisted of 15 boys and 5 girls. Although both groups had a higher proportion of boys, the difference in sex distribution was not statistically significant (Fisher’s exact test, *p* = 0.468). The mean age also did not differ significantly between groups (t = 1.58, *p* = 0.122). No significant eligible comorbidities were identified, indicating general comparability in baseline demographic characteristics (Table 1).

### 3.2. Weekly Changes in the Attention Composite and Eye-Tracking-Based Performance Metrics

**Figure 5:**
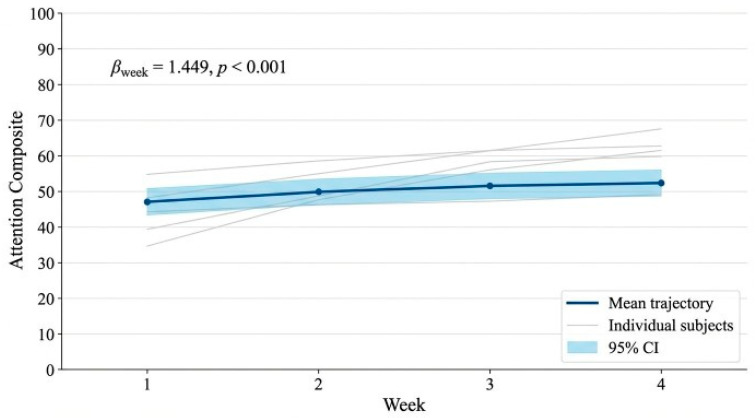
Weekly trajectory of the Attention Composite; weekly changes in the Attention Composite during the 4-week intervention period. The thick line represents the estimated mean trajectory derived from the linear mixed-effects model (LMM). The shaded area indicates the 95% confidence interval, and the thin lines represent individual participant trajectories.

Analysis of weekly changes in the primary outcome showed a gradual increase in Attention Composite scores over the 4-week intervention. The mean score increased from 47.63 in Week 1 to 50.04 in Week 2, 51.60 in Week 3, and 52.14 in Week 4 (Table 2, Figure 5). The largest increase occurred between Weeks 1 and 2, followed by a more gradual upward trend.

LMM results showed a significant main effect of time (β = 1.449, *p* < 0.001), indicating overall improvement in eye-tracking-based attentional performance. Random-effects estimates indicated variability in baseline performance and rates of change across participants.

**Figure 6:**
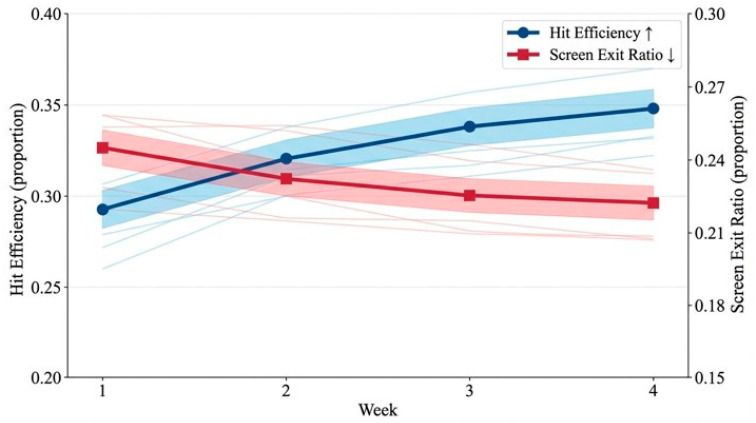
Weekly changes in eye-tracking metrics; weekly changes in eye-tracking-based performance metrics during the intervention period. The trajectories of hit efficiency and screen exit ratio across weeks are shown. Solid lines indicate mean values, shaded areas represent 95% confidence intervals, and thin lines indicate individual participant trajectories.

**Figure 7:**
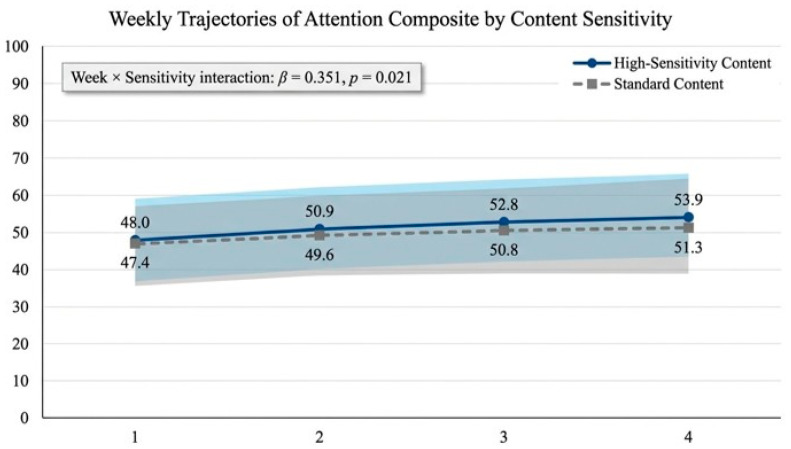
Weekly trajectories of the Attention Composite by content sensitivity; weekly changes in the Attention Composite according to content sensitivity. The solid blue line represents sessions using attention-sensitive content, and the gray dashed line represents sessions using standard content. Attention Composite scores showed a greater increase over time in the attention-sensitive condition, indicating a significant interaction between week and content sensitivity (β = 0.351, *p* = 0.021). Shaded areas indicate 95% confidence intervals.

Similar trends were observed in individual metrics. Hit efficiency gradually increased from 0.294 in Week 1 to 0.348 in Week 4, whereas the screen exit ratio decreased from 0.249 to 0.225 (Figure 6). These findings suggest that repeated exposure to the intervention improved task efficiency and reduced attentional disengagement. Additional analyses showed a significant interaction between time and content type (week × content sensitivity: β = 0.351, *p* = 0.021). As shown in Figure 7, attention-sensitive content increased from 48.0 in Week 1 to 53.9 in Week 4, whereas standard content increased from 47.4 to 51.3. Although both conditions improved over time, the attention-sensitive condition exhibited a steeper increase, suggesting an additional benefit of eye-tracking-based adaptive guidance.

### 3.3. Convergent Validity of Changes in the Attention Composite and CAT Scores

**Figure 8:**
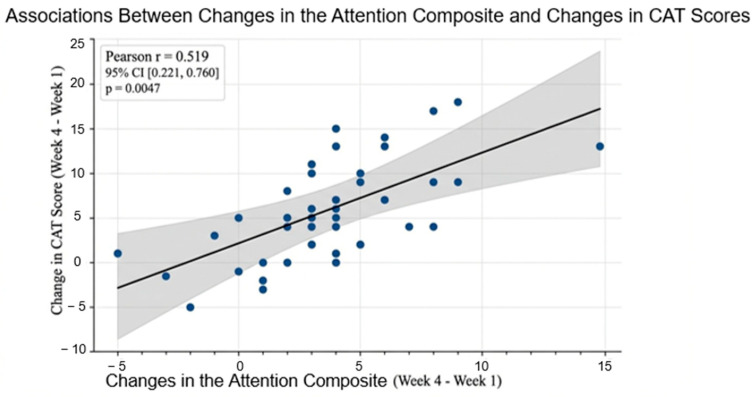
Associations between changes in the Attention Composite and CAT scores. The scatter plot illustrates the relationship between changes in the Attention Composite (Week 4 − Week 1) and changes in the Computerized Attention Test (CAT) scores. A positive correlation was observed, suggesting convergent validity between the eye-tracking-based Attention Composite and the standardized neuropsychological assessment. The shaded area represents the 95% confidence interval of the regression line.

To assess convergent validity, the correlation between changes in the Attention Composite from Week 1 to Week 4 (ΔAttention Composite) and CAT scores (ΔCAT) was examined. A statistically significant positive correlation was observed (r = 0.519, 95% bootstrap CI [0.221, 0.760], *p* = 0.0047) (Table 3, Figure 8). This indicates that improvements in the gaze-based metric were associated with improvements in the standardized measure of attentional function, suggesting that the Attention Composite may serve as a meaningful supplementary indicator. However, the moderate correlation suggests that the two measures capture related but non-identical constructs.

### 3.4. Stroop Color and Word Test Children’s Version

**Figure 9:**
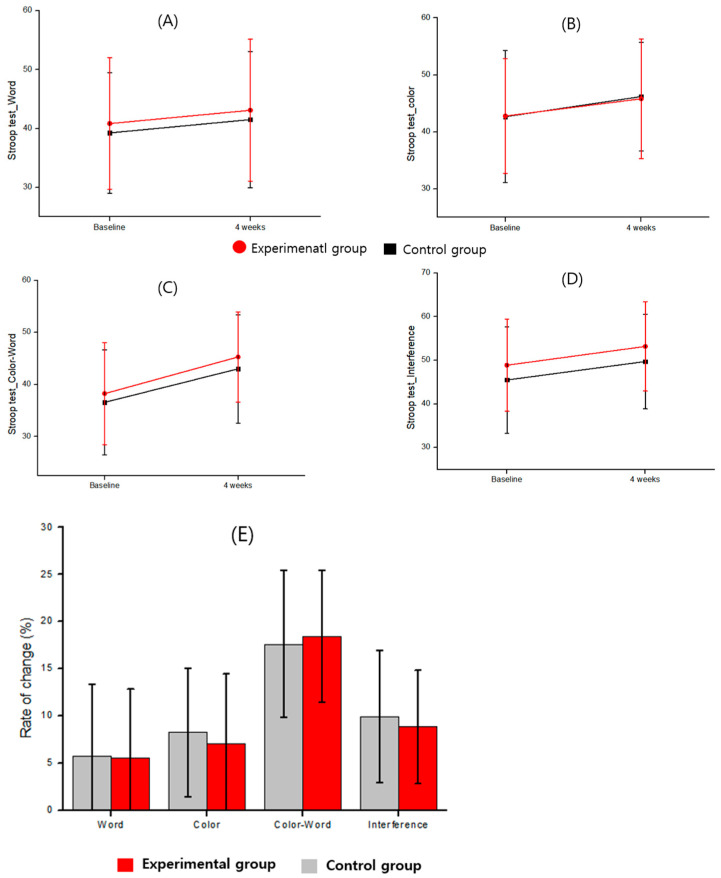
Changes in Stroop test scores and rate of change between baseline and 4 weeks. (**A**) Word, (**B**) Color, (**C**) Color–Word, (**D**) Interference, (**E**) Rate of Change.

Stroop test results are presented in Table 4 and Figure 9. In the Word and Color conditions, both groups showed slight score increases after four weeks; however, between-group differences were not statistically significant. These findings indicate that no clear differences were observed between the two intervention conditions in basic processing domains such as automated reading and color naming.

By contrast, more pronounced improvements were observed in the Color–Word condition, which more directly reflects selective attention and response inhibition. The experimental group increased from 38.26 ± 9.81 at baseline to 45.32 ± 8.65 at Week 4, with a significant within-group improvement (*p* = 0.002, Cohen’s d = 0.76). ANCOVA adjusting for baseline values showed significantly higher scores in the experimental group at Week 4 (*p* = 0.048, partial η^2^ = 0.10), corresponding to a moderate-to-large effect size.

A similar pattern was observed for the Interference scores. ANCOVA revealed a significant between-group difference (*p* = 0.021, partial η^2^ = 0.13), with higher scores in the experimental group at Week 4. These findings suggest that eye-tracking-based adaptive content selectively improved cognitive control, particularly response inhibition and selective attention, rather than overall processing speed.

### 3.5. K-WISC-V Processing Speed Subtest Outcomes

**Figure 10:**
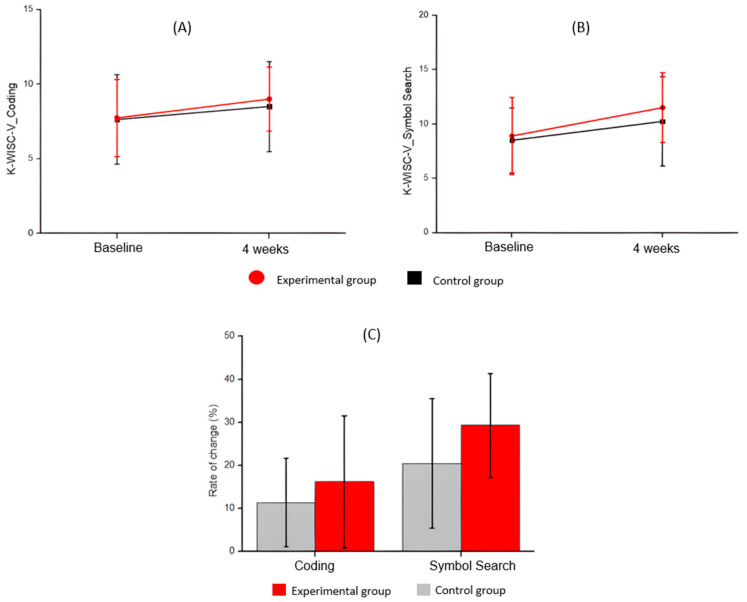
Changes in K-WISC-V subtest scores (Coding, Symbol Search) and rate of change between groups; (**A**) Coding score change, (**B**) Symbol Search score change, (**C**) comparison of rate of change.

K-WISC-V processing speed results are presented in Table 5 and Figure 10. In the Coding subtest, both groups showed score increases after 4 weeks; however, the between-group difference was not statistically significant after adjusting for baseline values (ANCOVA, *p* = 0.085), suggesting no consistent effect across all processing speed measures.

By contrast, greater improvements were observed in the Symbol Search subtest in the experimental group. Scores increased from 8.91 ± 3.54 at baseline to 11.52 ± 3.20 at Week 4, compared with 8.51 ± 3.02 to 10.25 ± 4.10 in the control group. Although a direct comparison at Week 4 did not show a significant difference (*p* = 0.283), ANCOVA adjusting for baseline values revealed a significant between-group difference (*p* = 0.034, partial η^2^ = 0.11). Percentage change analysis further indicated a greater improvement in the experimental group (29.30%) than in the control group (20.45%) (*p* = 0.040, Cohen’s d = 0.65).

These findings suggest that eye-tracking-based adaptive content was associated with greater improvements in tasks requiring visual search and target detection than in general processing speed measures.

At the neuropsychological level greater improvements in the experimental group were observed in the Stroop Color–Word and Interference scores and the K-WISC-V Symbol Search subtest. By contrast, no clear between-group differences were observed in more basic measures such as Word, Color, and Coding. These findings suggest that the intervention selectively influenced core ADHD-related cognitive functions, such as selective attention, interference control, and visual search, rather than producing generalized performance improvements.

## 4. Discussion

This study examined the feasibility and preliminary effects of an eye-tracking-based interventional learning guide in a self-administered digital therapeutic environment for children with ADHD. First, the Attention Composite significantly increased over the 4-week intervention. Hit efficiency increased, and the screen exit ratio decreased, indicating improved task efficiency and reduced attentional disengagement. Second, attention-sensitive content demonstrated a steeper improvement trajectory than standard content, suggesting added benefit from eye-tracking-based adaptive guidance. Third, changes in the Attention Composite were significantly positively correlated with CAT score changes, supporting convergent validity. Fourth, selective improvements were observed in the Stroop Color–Word and Interference scores and the K-WISC-V Symbol Search subtest, suggesting more direct effects on specific cognitive domains.

These preliminary patterns are consistent with selective effects on core ADHD-related cognitive functions rather than generalized processing speed improvements. Consistent with this interpretation, no clear between-group differences were observed in basic processing measures such as the Stroop Word and Color conditions and the K-WISC-V Coding subtest. By contrast, greater improvements were observed in the Stroop Color–Word and Interference conditions and the K-WISC-V Symbol Search subtest, which reflect response inhibition, interference control, and target detection. These results suggest that eye-tracking-based learning guidance may be associated with improvements in the cognitive control processes involved in maintaining attention to task-relevant stimuli and inhibiting distractors, rather than simply increasing task engagement [53].

The present intervention was conceptually grounded in attentional regulation and cognitive control models of ADHD. Real-time gaze monitoring was used to detect attentional disengagement during cognitively demanding tasks, and adaptive feedback was designed to facilitate task re-engagement and sustained attentional control.

The greater improvement trajectory observed in attention-sensitive content further suggests that the effectiveness of DTx may depend not only on eye-tracking functionality but also on how sensitively attentional disengagement is detected and how promptly feedback is delivered [54].

These findings are clinically relevant because they frame the effectiveness of ADHD DTx not only in terms of content-driven training effects but also in terms of adherence and underlying mechanisms, particularly the detection of attentional disengagement and real-time guidance during self-administered use. This highlights the importance of closed-loop intervention systems that detect disengagement and redirect attention to task-relevant stimuli [55,56]. In other words, this study further supports the potential of eye-tracking not only as an assessment tool but also as a complementary mechanism for adaptive therapeutic intervention within digital therapeutic environments [57]. These findings extend prior gaze-contingent and adaptive feedback approaches by applying real-time gaze-based guidance within a self-administered ADHD digital therapeutic setting. In home-based settings, where immediate guidance from clinicians or caregivers is limited, such adaptive systems may improve real-world applicability and sustainability.

The significant correlation between Attention Composite changes and CAT scores further supports this interpretation. Although the correlation was moderate, suggesting that the measures capture related but non-identical constructs, the findings indicate that the gaze-based metrics are not merely device-generated logs but reflect cognitive changes that are partially shared with standardized measures of attention [58]. Therefore, the Attention Composite may serve as a complementary outcome measure in future ADHD digital therapeutic studies, particularly for understanding adherence, performance processes, and mechanisms of change. While the moderate correlation with CAT scores (r = 0.519) provides preliminary evidence of convergent validity, discriminant validity and broader construct validity of the Attention Composite remain to be established. These psychometric properties should be systematically evaluated in future studies with larger and more diverse samples.

Digital therapeutic interventions used in self-administered environments may have several limitations, including reduced adherence, attentional disengagement, mechanical responding, fatigue associated with screen use, variability in eye-tracking accuracy, privacy concerns, and limited generalizability to real-world functioning. The eye-tracking-based adaptive learning guide proposed in the present study was designed to address some of these limitations, particularly by detecting and redirecting attentional disengagement in real time; however, it does not fully resolve all limitations associated with digital therapeutics. Therefore, the findings of this study should be interpreted cautiously as preliminary evidence supporting the potential of self-administered ADHD digital therapeutics to improve attentional performance, rather than as confirmatory evidence of long-term clinical efficacy.

This study had several limitations. First, as a pilot study with a limited sample size, the findings should be interpreted as preliminary and exploratory, reflecting preliminary efficacy rather than confirmatory evidence. Second, the relatively short intervention period of four weeks makes it difficult to determine the durability of the effects, long-term clinical symptom improvement, and the transfer of effects to real-world functioning. Third, because participants continued pharmacological treatment, the independent effects of the digital intervention could not be isolated. The observed effects may reflect a combined or synergistic influence of medication and the digital intervention. In addition, although gaze-based metrics demonstrated meaningful results, further psychometric validation and clinical utility should be established in larger samples with longer follow-up. Furthermore, the Attention Composite is an internally developed measure for which formal reliability estimates, including internal consistency and test–retest reliability, have not yet been established. The consistent weekly improvement trajectory observed across sessions may provide indirect evidence of score stability; however, psychometric validation in larger samples is necessary before this measure can be adopted as a standardized outcome in future studies.

Fourth, because the present system relied on RGB camera-based gaze estimation, excessive head movement, blinking behavior, and environmental lighting variability may have influenced gaze-estimation stability, particularly in children with ADHD. However, the system was designed to detect broad attentional engagement and disengagement patterns during self-administered digital therapeutic use rather than perform microsaccade-level gaze localization or ophthalmologic fixation analysis. To improve robustness under ecologically realistic usage conditions, preprocessing procedures, invalid-frame exclusion, and recalibration workflows were applied to reduce major tracking instability and motion-related artifacts. In addition, as the gaze estimation system excluded approximately 14.7% of frames due to tracking instability, residual measurement error may have introduced variability into the Attention Composite scores. The potential influence of this measurement error on score precision cannot be fully excluded and should be considered when interpreting the findings. Future studies using larger samples and additional validation against laboratory-grade eye-tracking systems may further improve technical reliability and generalizability. Furthermore, the relatively large number of statistical comparisons conducted in this study raises concerns regarding potential Type I error inflation. As no formal correction for multiple comparisons was applied, given the exploratory nature of the study, statistically significant findings should be interpreted cautiously and regarded as preliminary rather than confirmatory. Future studies should employ pre-registered hypotheses and appropriate correction procedures to reduce the risk of false-positive findings. Finally, because the experimental and control interventions were implemented using different software platforms, potential platform-specific effects on engagement, motivational characteristics, and user experience cannot be completely excluded, despite matching therapeutic targets and task structures. Future studies employing a within-platform design, in which eye-tracking guidance is selectively enabled or disabled within the same software environment, would allow for more precise isolation of the eye-tracking component’s contribution.

Overall, this study suggests that an eye-tracking-based interventional learning guide may improve attentional performance in self-administered digital therapeutics for children with ADHD and provide selective benefits in domains related to selective attention and cognitive control. These findings provide preliminary evidence that DTx for ADHD may evolve into adaptive intervention platforms that respond to moment-to-moment attentional states.

## 5. Conclusions

In children with ADHD, digital therapeutic content incorporating an eye-tracking-based interventional learning guide significantly improved the primary outcome, the Attention Composite, from 47.63 in Week 1 to 52.14 in Week 4 (β = 1.449, *p* < 0.001). The attention-sensitive condition showed a greater improvement trajectory than the standard condition (48.0→53.9 vs. 47.4→51.3; β = 0.351, *p* = 0.021). Furthermore, changes in the Attention Composite were positively correlated with CAT score changes (r = 0.519, 95% bootstrap CI [0.221, 0.760], *p* = 0.0047), supporting convergent validity.

Among the secondary outcomes, significant between-group differences were observed in the Stroop Color–Word condition (ANCOVA, *p* = 0.048, partial η^2^ = 0.10), Interference score (ANCOVA, *p* = 0.021, partial η^2^ = 0.13), and the K-WISC-V Symbol Search subtest (ANCOVA, *p* = 0.034, partial η^2^ = 0.11; percentage change 29.30% vs. 20.45%, *p* = 0.040), whereas no significant differences were found in the Stroop Word and Color conditions or the K-WISC-V Coding subtest.

These findings suggest that self-administered ADHD DTx incorporating eye-tracking-based adaptive guidance may improve attentional performance and provide selective benefits in selective attention, interference control, and visual search. However, these findings remain preliminary and should not be interpreted as confirmatory evidence of clinical efficacy. Further large-scale studies with longer follow-up are required to confirm these findings.

## Figures and Tables

**Table 1:** Information on participating children.

		Experimental Group (N = 20)		Control Group (N = 20)		Accompanying Symptoms	*p*-Value
		Mean or N	SD or %	Mean of N	SD or %		0.468 *
Gender	Male	17	85	15	75	None	
	Female	3	15	5	25	None	
Age		Mean	SD	Mean	SD		0.122 **
		9.02	0.56	8.63	1.86		

* Fisher’s exact test; ** independent *t*-test.

**Table 2:** Weekly changes in the Attention Composite and eye-tracking metrics.

Week	Attention Composite (Mean)	Hit Efficiency (Mean)	Screen Exit Ratio (Mean)
Week 1	47.63	0.294	0.249
Week 2	50.04	0.324	0.237
Week 3	51.60	0.339	0.225
Week 4	52.14	0.348	0.225

Values represent mean scores calculated from session-level data. Attention Composite range: 0–100. Eye-tracking metrics range: 0–1.

**Table 3:** Correlations between changes in the Attention Composite and CAT scores.

Variable	Pearson r	95% Bootstrap CI	*p*-Value
∆Composite vs. ∆CAT	0.519	[0.221, 0.760]	0.0047

**Table 4:** Stroop test results.

			Experimental Group (N = 20)		Control Group (N = 20)		*p*-Value	Effect Size
			Mean	SD	Mean	SD		
Stroop test	Word	Baseline	40.84	11.21	39.25	10.25	0.582 *	
		4 weeks	43.10	12.05	41.51	11.59	0.620 *	
		*p*-value	0.082 *		0.254 *		0.410 ***	0.02 ##
		Effect size	0.19 #		0.30 #			
		Rate of change (%)	5.53	7.30	5.76	7.60	0.450 *	0.03 #
	Color	Baseline	42.79	10.04	42.67	11.58	0.241 *	
		4 weeks	45.81	10.48	46.20	9.52	0.620 *	
		*p*-value	0.061 *		0.102 *		0.310 ***	0.03 ##
		Effect size	0.29 #		0.33 #			
		Rate of change (%)	7.06	7.40	8.27	6.80	0.620 *	0.17 #
	Color–word	Baseline	38.26	9.81	36.58	10.09	0.420 *	
		4 weeks	45.32	8.65	43.02	10.41	0.521 *	
		*p*-value	0.002 *		0.101 *		0.048 ***	0.10 ##
		Effect size	0.76 #		0.63 #			
		Rate of change (%)	18.45	7.00	17.60	7.80	0.042 *	0.12 #
	Interference Score	Baseline	48.89	10.56	45.25	12.20	0.780 *	
		4 weeks	53.20	10.25	49.75	10.85	0.561 *	
		*p*-value	0.420 *		0.020 *		0.021 **	0.13 ##
		Effect size	0.41 #		0.39 #			
		Rate of change (%)	8.82	6.00	9.94	7.00	0.051 *	0.17 #

* Independent *t*-test; ** baseline vs. 4 weeks paired *t*-test; *** baseline-adjusted ANCOVA (analysis of covariance); # Cohen’s d; ## partial η^2^.

**Table 5:** Korean-Wechsler Intelligence Scale for Children (K-WISC-V).

			Experimental Group (N = 20)		Control Group (N = 20)		*p*-Value	Effect Size
			Mean	SD	Mean	SD		
K-WISC-V	Coding	Baseline	7.75	2.58	7.64	3.01	0.180 *	
		4 weeks	9.01	2.15	8.51	3.02	0.2018 *	
		*p*-value	0.512 **		0.045 **		0.085 ***	0.075 ^##^
		Effect size	0.53 ^#^		0.29 ^#^			
		Rate of change (%)	16.26	15.31	11.39	10.25	0.420 *	0.37 ^#^
	Symbol Search	Baseline	8.91	3.54	8.51	3.02	0.520 *	
		4 weeks	11.52	3.20	10.25	4.10	0.283 *	
		*p*-value	0.210 **		0.105 **		0.034 ***	0.11 ^##^
		Effect size	0.77 ^#^		0.48 ^#^			
		Rate of change (%)	29.30	12.04	20.45	15.02	0.040 *	0.65 ^#^

* Independent *t*-test; ** baseline vs. 4 weeks paired *t*-test; *** baseline-adjusted ANCOVA (analysis of covariance); ^#^ Cohen’s d; ^##^ partial η^2^.

## Data Availability

The original contributions presented in the study are included in the article, further inquiries can be directed to the corresponding authors.

## Abbreviations

The following abbreviations are used in this manuscript:

ADHD	Attention-Deficit/Hyperactivity Disorder
DSM-5	Diagnostic and Statistical Manual of Mental Disorders, Fifth Edition
DTx	Digital Therapeutics
AOI	Area of Interest
IRB	Institutional Review Board
CRIS	Clinical Research Information Service
CGI-S	Clinical Global Impression–Severity
K-WISC-V	Korean Wechsler Intelligence Scale for Children–Fifth Edition
PSI	Processing Speed Index
LMM	Linear Mixed-Effects Model
ANCOVA	Analysis of Covariance
REML	Restricted Maximum Likelihood
CAT	Comprehensive Attention Test
